# Dual‐Color‐Emitting Carbon Nanodots for Multicolor Bioimaging and Optogenetic Control of Ion Channels

**DOI:** 10.1002/advs.201700325

**Published:** 2017-10-03

**Authors:** Hyemin Kim, Yoonsang Park, Songeun Beack, Seulgi Han, Dooyup Jung, Hyung Joon Cha, Woosung Kwon, Sei Kwang Hahn

**Affiliations:** ^1^ PHI Biomed Co., #613 12 Gangnam‐daero 65‐gil Seocho‐gu Seoul 06612 South Korea; ^2^ Department of Chemical Engineering Pohang University of Science and Technology (POSTECH) 77 Cheongam‐ro Nam‐gu, Pohang Gyeongbuk 37673 South Korea; ^3^ Department of Materials Science and Engineering Pohang University of Science and Technology (POSTECH) 77 Cheongam‐ro Nam‐gu, Pohang Gyeongbuk 37673 South Korea; ^4^ Department of Chemical and Biological Engineering Sookmyung Women's University 100 Cheongpa‐ro 47‐gil Seoul 04310 South Korea

**Keywords:** carbon nanodots, multicolor bioimaging, optogenetics, surface modification

## Abstract

The development of intrinsically multicolor‐emitting carbon nanodots (CNDs) has been one of the great challenges for their various fields of applications. Here, the controlled electronic structure engineering of CNDs is performed to emit two distinct colors via the facile surface modification with 4‐octyloxyaniline. The so‐called dual‐color‐emitting CNDs (DC‐CNDs) can be stably encapsulated within poly(styrene‐*co*‐maleic anhydride) (PSMA). The prepared water‐soluble DC‐CNDs@PSMA can be successfully applied to in vitro and in vivo dual‐color bioimaging and optogenetics. In vivo optical imaging can visualize the biodistribution of intravenously injected DC‐CNDs@PSMA. In addition, the light‐triggered activation of ion channel, channelrhodopsin‐2, for optogenetic applications is demonstrated. As a new type of fluorophore, DC‐CNDs offer a big insight into the design of charge‐transfer complexes for various optical and biomedical applications.

Carbon nanodots (CNDs) have been widely investigated as new multifunctional nanoparticles for various applications ranging from optoelectronic devices to theranostic agents.^1^, ^2^, ^3^, ^4^ CNDs have a great potential as biocompatible optical materials that can be easily prepared from abundant carbon sources through facile synthetic methods. It has been known that the optical property of CNDs largely depends upon their surface states, or more specifically, their functional groups, the defect density, and the presence of adsorbates on the surface of CNDs.^5^ Accordingly, in the past decade, many research efforts have been devoted for modifying and controlling the surface characteristics of CNDs to expand their applicability.^6^, ^7^, ^8^, ^9^, ^10^ In this context, for instance, the photoluminescence (PL) wavelength could be controlled via the energy‐level engineering,^6^, ^7^ the ion selectivity could be endowed for detecting metal ions,^8^ and the biocompatibility could be improved for biomedical applications.^9^, ^10^, ^11^ On the basis of these unique characteristics,^12^, ^13^ CNDs have been exploited for a variety of biomedical applications, such as diagnostic applications,^14^ photoacoustic imaging, and the photothermal therapy.^15^, ^16^, ^17^

Recently, multicolor PL nanomaterials have become greatly important in cell counting, cell sorting, diagnostics, and other therapeutic applications.^18^ In flow cytometry, a single multicolor agent can be used with multiple excitation and emission wavelengths for diverse commercial instruments. Furthermore, these multicolor agents show the stimuli responsive behaviors, enabling the applications to the multimodal sensing.^19^ With the progress of light‐guided therapies, multicolor PL nanomaterials are also expected to be used in various types of phototherapies including photodynamic therapy and optogenetic therapy. The optogenetics can be simply defined as the use of light to control cells in the living tissue. In accordance, the development of multicolor‐emitting CNDs becomes very important to expand the capabilities of CNDs for biomedical applications. So far, the tuning of PL colors of CNDs has been extensively investigated, but only few studies have reported the “intrinsic” multicolor PL.^20^, ^21^, ^22^ The multicolor PL represents two or more distinct and totally independent PL colors generated from possibly different origins in contrast to the conventional excitation‐light‐dependent PL reported elsewhere.^3^, ^23^

Here, we developed dual‐color‐emitting CNDs (DC‐CNDs) by controlling the electronic structure of CNDs through a facile surface modification with 4‐octyloxyaniline (**Figure** **1**a). DC‐CNDs could be stably encapsulated within water‐soluble poly(styrene‐*co*‐maleic anhydride) (PSMA), enabling biomedical applications to in vitro and in vivo dual‐color bioimaging and light‐triggered activation of ion channel for optogenetic applications (Figure 1b). First, we have carried out in vitro cellular imaging with DC‐CNDs by confocal and two‐photon microscopy. In addition, we have visualized in vivo distribution of intravenously injected DC‐CNDs. Furthermore, we have demonstrated that two distinct PL of DC‐CNDs could be used for selective activation of channelrhodopsin‐2 (ChR2), a light‐switched cation channel, reflecting the feasibility for optogenetic applications.

**Figure 1:**
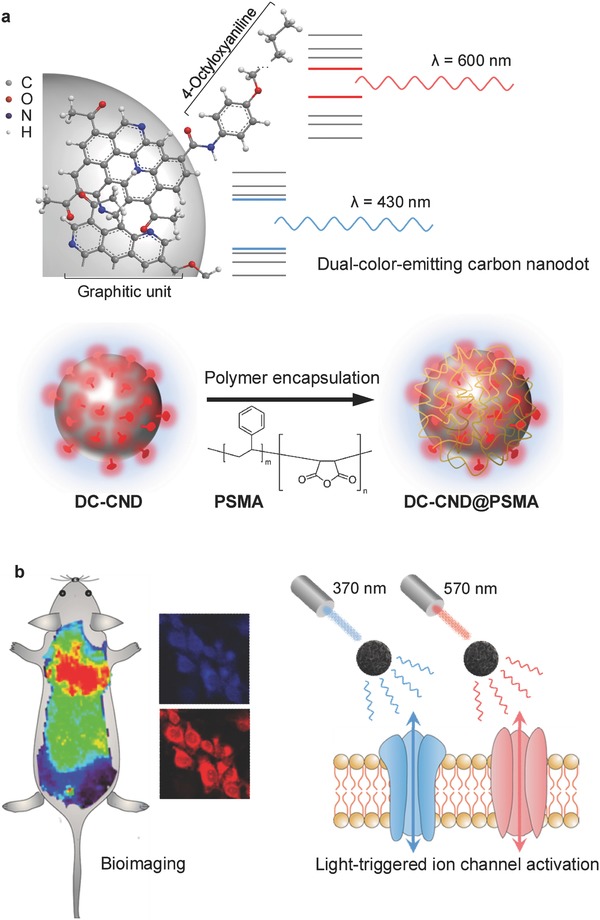
a) Schematic representation for the chemical structure of carbon nanodots (CNDs) showing the dual‐color‐emitting (DC) property (top) and the encapsulation of DC‐CNDs into poly(styrene‐*co*‐maleic anhydride) (DC‐CNDs@PSMA) (bottom). b) Schematic illustration for the applications of facile DC‐CNDs to the fields of bioimaging and optogenetics.

The pristine CNDs were prepared by the solvothermal carbonization of citric acid.^24^ Then, the pristine CNDs were subject to surface modification with 4‐octyloxyaniline to prepare DC‐CNDs (2–3 nm in size, **Figure** **2**a).^25^ The degree of surface modification was carefully controlled by changing the concentration of 4‐octyloxyaniline. The as‐prepared DC‐CNDs then formed water‐soluble composites with PSMA (DC‐CNDs@PSMA) through hydrophobic interaction between the octyl group of DC‐CNDs and the styrene group of PSMA (Figure S1, Supporting Information). The transmission electron microscopy (TEM) showed that DC‐CNDs@PSMA was well‐dispersed with a particle size of 3–6 nm (Figure 2b). The DC‐CNDs@PSMA appeared to be composed of one to four DC‐CNDs (Figure S2, Supporting Information). To investigate the chemical structure of DC‐CNDs, we performed X‐ray photoelectron spectroscopy (XPS) and ^13^C nuclear magnetic resonance (NMR) analyses. The deconvoluted C1s XPS spectra indicated the presence of C—C/C=C (284.5 eV), C—N (285.9 eV), and C=O (288 eV) bondings on the surface of DC‐CNDs, likely due to oxidative carbonization (Figure 2c). ^13^C NMR was performed to analyze the surface chemistry of pristine CNDs and DC‐CNDs (Figure S3, Supporting Information). In Figure S3a (Supporting Information), pristine CNDs showed chemical shifts of 14.1, 22.7, 29.6, 31.9, 32.6 (alkyl) and 129.7, 129.9 ppm (alkene) for the oleyl group, and 33.8 ppm for amido carbon, indicating that oleylamine was passivated on the surface of CNDs. After modification with 4‐octyloxyaniline, DC‐CNDs showed peaks at around 68.3, 132.3, and 158.6 ppm for methoxy, anilino, and phenoxy groups, together with the oleyl peaks, respectively (Figure S3b, Supporting Information). The results revealed the presence of partially substituted 4‐octyloxyanilines on the surface of DC‐CNDs. The ^1^H NMR spectrum of DC‐CNDs@PSMA in Figure S3c (Supporting Information) showed the chemical shifts at 1.2, 1.8 (alkyl), 2.2 (arene‐CH), 3.1 (HO—CH), 3.3 (O=C—NH), and 7.1 ppm (arene‐H), indicating the presence of PSMA on the surface of DC‐CNDs. The surface functionalities of DC‐CNDs have been further identified by the Fourier‐transform infrared spectroscopy (Figure S4, Supporting Information). Both pristine CNDs and DC‐CNDs exhibited the C=O stretching (1690 cm^−1^) and N—H bending (1500 cm^−1^) peaks, confirming the presence of carbonyl and amide (from oleylamine) groups on the surface of CNDs. After surface modification, the N—H stretching (3500–3300 cm^−1^) and C—O stretching (1260 cm^−1^) peaks were developed and the N—H bending and C=C stretching (1600–1450 cm^−1^) bands were intensified due to the chemical bonding of 4‐octyloxyaniline to the surface of CNDs.

**Figure 2:**
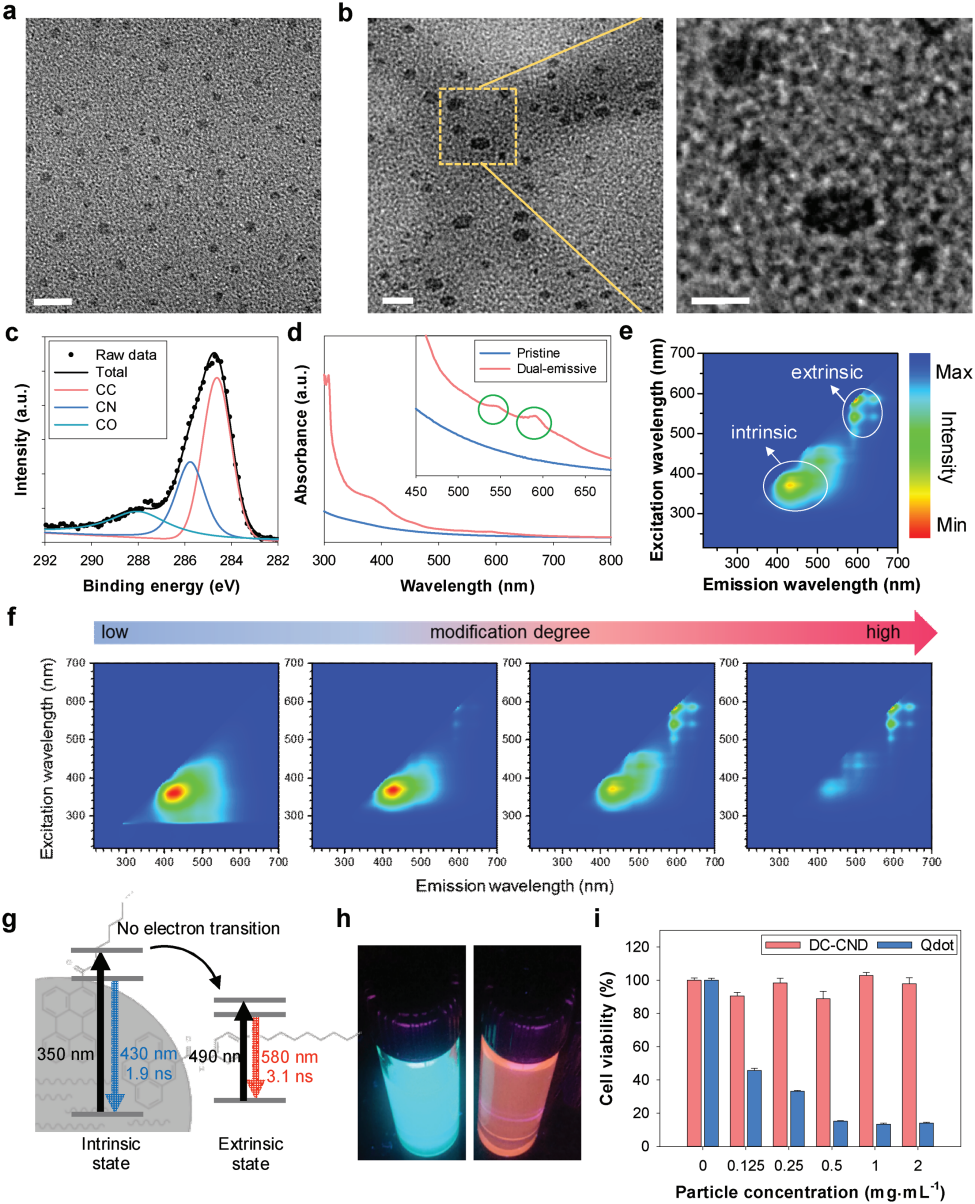
a) TEM images of dual‐color‐emitting carbon nanodots (DC‐CNDs, scale bar = 10 nm). b) TEM images of poly(styrene‐*co*‐maleic anhydride) (PSMA) encapsulated DC‐CNDs (left). The dotted area in the middle panel is magnified in the right panel (scale bar = 10 nm for left and 5 nm for right). c) Deconvoluted carbon (1s) XPS spectra of DC‐CNDs. d) The absorption spectra of DC‐CNDs. The inset is the expansion from 400 to 700 nm. The PL maps of e) DC‐CNDs and f) the changes of PL as a function of surface functionalization degree controlled by varying the concentration of 4‐octyloxyaniline. The used solvent was toluene. g) The emission structure of DC‐CNDs. h) The photoimage of DC‐CNDs under the illumination of 350 (left) or 600 nm (right) light. (i) The cytotoxicity of DC‐CNDs@PSMA and quantum dots (Qdots) in FL83B cells.

The electronic structure and optical property of DC‐CNDs were investigated by UV–vis absorption spectroscopy and PL spectroscopy. Figure 2d shows the absorption spectra of pristine CNDs and DC‐CNDs. Because both pristine CNDs and DC‐CNDs showed two strong absorption peaks at around 300 and 350 nm in common, the energy states related to the aforementioned absorption peaks might be called “intrinsic” energy states. The first peak at 300 nm could be assigned to π–π* transitions that take place in the carbonized cores of CNDs. Because the π–π* gap of 5–8 fused benzene rings is 4–5 eV,^26^, ^27^ the core of CNDs might be composed of a few angstrom‐sized polyaromatic carbon domains surrounded by the amorphous carbon matrix. The second peak at 350 nm might be assigned to n–π* transitions between the n‐orbitals of heteroatoms (nitrogen and oxygen) and the π* orbitals of the polyaromatic domains. This presumably indicated that CNDs contained a significant amount of nitrogen and oxygen atoms originated from nitric acid as an oxidative carbonization catalyst and oleylamine as a surface passivation agent, respectively (Figure S5 and Table S1, Supporting Information). In comparison with pristine CNDs, DC‐CNDs exhibited a significant absorption peak at 590 nm, indicating that a new energy state was developed by the surface modification, so‐called the “extrinsic” energy state. Due to the small size of CNDs only in the range of 2–3 nm, most of their polyaromatic domains would be exposed to the exterior. Accordingly, they can be readily modified with 4‐octyloxyaniline to form the extrinsic energy states.^25^ The newly observed absorption showed the characteristics of n–π* transition, such as visible‐range excitation and low extinction coefficient.^24^, ^28^ In Figure 2e, the PL data of DC‐CNDs show two clearly discernible PL emissions with almost no spatial overlap at the wavelengths of 430 nm (blue, 370 nm excitation) and 600 nm (red, 570 nm excitation). By comparing the PL spectra of the pristine CNDs and DC‐CNDs (Figure S6, Supporting Information), it could be confirmed that the blue PL and the red PL were originated from intrinsic energy states and from extrinsic energy states, respectively. To investigate the reason for two distinct PL emissions, the degree of surface modification was controlled by changing the concentration of 4‐octyloxyaniline. With increasing modification, the intrinsic PL band disappeared, but the extrinsic PL peak increased significantly (Figure 2f). These results were matched well with the chemical structure of our CNDs described above. The polyaromatic domains responsible for intrinsic PL in the core of our CNDs would be converted to 4‐octyloxyaniline‐modified polyaromatic domains responsible for extrinsic PL with the surface modification (Figure S7, Supporting Information). Furthermore, we examined the PL spectra of DC‐CNDs before and after the PSMA encapsulation. As shown in Figure S8 (Supporting Information), the PSMA did not significantly change the optical properties of DC‐CNDs, because the hydrophobic chains of PSMA protected the chemical structures of DC‐CNDs. There were only a slight red‐shift (≈5 nm) and the broadening of PL presumably due to the polarity change in surroundings induced by the PSMA. The quantum yield of DC‐CNDs was ≈30% at 550 nm excitation, which was considered as the highest record among red‐color‐emissive CNDs (Figure S9, Supporting Information).^25^, ^29^, ^30^

In order to explore the energy structure of DC‐CNDs, we conducted time‐resolved absorption (TA) spectroscopy and time‐resolved PL (TRPL) spectroscopy. TA spectra were recorded to show the electronic transition in intrinsic and extrinsic states at the probe wavelength of 580 nm under the excitation wavelengths of 350 and 540 nm, respectively. Each of them corresponds to the transition energy of intrinsic and extrinsic states (Figure S10, Supporting Information). By the excitation at 350 nm, DC‐CNDs exhibited excited state absorption (∆*A* > 0) on all timescales. The photoexcited electrons from intrinsic states were consumed to other accessible states, but were not allowed to access extrinsic states, indicating that the intrinsic states were localized in the polyaromatic domains and isolated from the 4‐octyloxyaniline‐modified polyaromatic domains. By the excitation at 540 nm, ∆*A* values showed strong stimulated emission (∆*A* < 0) even after 1 ns time delay. The photoexcited electrons from extrinsic states might be directly related to the red emission (580 nm). The TA spectra were fitted using biexponential functions (Table S2, Supporting Information). The relatively long decay time by the excitation at 540 nm might be attributed to the molecule‐like, defectless characteristics of extrinsic states.^25^

To investigate the dynamics of radiative recombination of DC‐CNDs, TRPL spectra were recorded at the probe wavelengths of 430 and 580 nm, each of which corresponds to the emission wavelengths from intrinsic and extrinsic states (Figure S11 and Table S3, Supporting Information). The TRPL spectra were well fitted to single exponential functions, reflecting that each emission state had one dominant radiative recombination pathway. The PL lifetime of extrinsic emission (3.1 ns) was longer than that of intrinsic emission (1.9 ns) in consistent with the TA decay time data. Furthermore, the PL lifetime of intrinsic emission of DC‐CNDs (2.0 ns) was analogous to that of the pristine CNDs (2.4 ns) (Figure S12 and Table S4, Supporting Information), reflecting that there was no relaxation pathway of photoexcited electrons from intrinsic energy states to extrinsic energy states. From TA and TRPL spectra analysis, the electronic structure of DC‐CNDs was schematically represented in Figure 2g. The important feature was that there was no energy transfer between intrinsic and extrinsic states, even the physical distance between the two states were very close to each other, probably less than the size of DC‐CNDs (3 nm). Three reasons might be proposed for no energy transfer: (i) a significant energy difference, (ii) the insulating amorphous carbon matrix acting as a barrier for electronic transitions, and (iii) the perpendicular transition dipoles.^31^

For further biomedical applications, the cytotoxicity of DC‐CNDs was evaluated in the normal hepatocyte of FL83B cells (Figure 2i). The hepatocytes were incubated for 24 h with DC‐CNDs@PSMA dispersed in the cell culture media at the concentration range of 0.125–2 mg mL^−1^. DC‐CNDs showed high cytocompatibility with a cell viability over 88% even at the high concentration of 2 mg mL^−1^. As a control group, we used commercially available CdSe/ZnS quantum dots coated with a polymer bearing carboxyl groups, which showed high toxicity at the same concentrations. The low cytotoxicity of DC‐CNDs@PSMA was also confirmed in several different kinds of normal cells like embryonic kidney cells (HEK293) as well as cancer cells like hepatocellular carcinoma cells (HepG2) and skin melanoma cells (B16F10) (Figure S13, Supporting Information).

In vitro cellular imaging was carried out in a skin melanoma cell of B16F10 cells to assess the performance of DC‐CNDs as a bioimaging agent. After treatment with DC‐CNDs@PSMA in the cell culture media at a concentration of 0.5 mg mL^−1^ for 1 h, DC‐CNDs@PSMA appeared to be internalized into the cells, showing the bright and robust fluorescence emission (**Figure** **3**a). According to the confocal microscopic imaging, the cells labeled with DC‐CNDs@PSMA showed different emission colors depending on the excitation wavelength. DC‐CNDs emitted blue fluorescence with excitation at 405 nm and red fluorescence with 561 nm. Because DC‐CNDs showed high two‐photon excitation, we could demonstrate their feasibility as a two‐photon imaging agent. With illumination of two‐photon laser at 950 nm, DC‐CNDs showed bright yellow and red colors. Furthermore, in HEK293, FL83B, and HepG2, DC‐CNDs@PSMA was also readily internalized into the cells and emitted strong fluorescence, regardless of the shape and malignance of the cells (Figure S14, Supporting Information).

**Figure 3:**
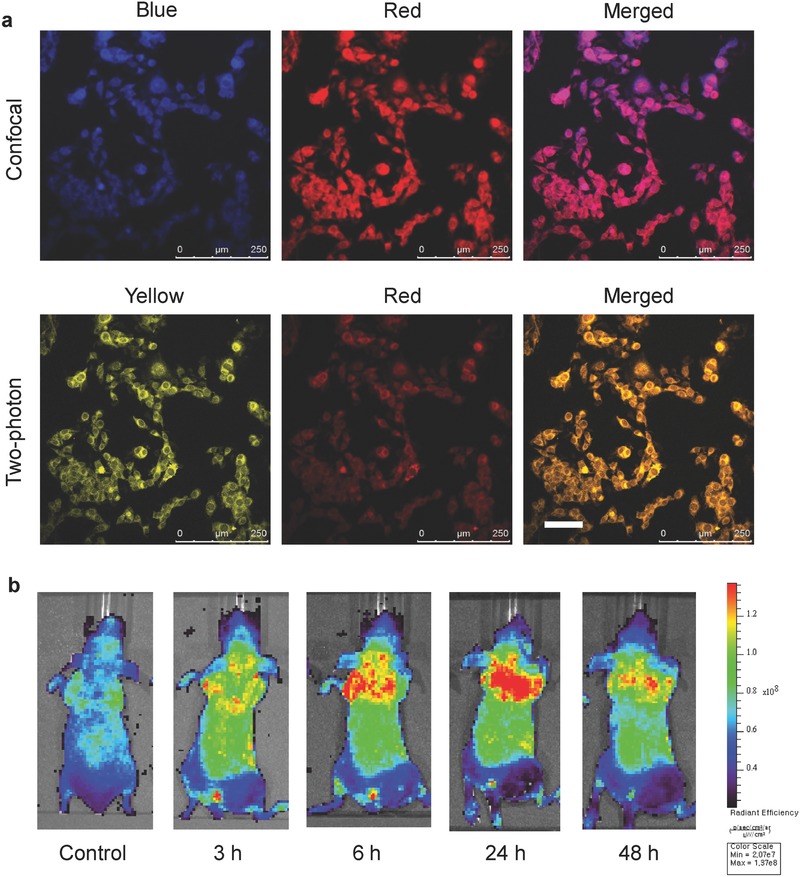
a) Multicolor confocal (top) and two‐photon (bottom) microscopic images of B16F10 cells after treatment of dual‐color‐emitting carbon nanodots encapsulated in poly(styrene‐*co*‐maleic anhydride) (DC‐CNDs@PSMA) (scale bar = 100 µm). b) In vivo optical bioimaging after intravenous injection of DC‐CNDs@PSMA to mice.

With a distinct absorption peak at 590 nm, DC‐CNDs have been used for in vivo optical imaging to track the biodistribution and clearance of DC‐CNDs@PSMA. The intravenously injected DC‐CNDs@PSMA circulated and accumulated throughout the whole body, showing the bright fluorescence signal mainly in the lung. The fluorescence signal was the strongest at 24 h postinjection and then gradually decreased due to clearance. The surface encapsulation of nanoparticles with hydrophilic polymers has been known to mitigate the interaction of nanoparticles with macrophages in the mononuclear phagocytic system, enhancing the circulation of the nanoparticles due to the reduced immune clearance.^32^, ^33^ The PSMA has been known to prolong the plasma half‐life of conjugated molecules by binding to the serum albumin (ALB) in blood.^34^ DC‐CNDs@PSMA with an increased particle size also showed a relatively long circulation time compared with other CNDs reported elsewhere.^35^

Inspired by two different excitation and emission centers (Figure 2), we carried out a proof‐of‐concept study of DC‐CNDs for optogenetic applications. Two different ion channels of neurons were hypothesized to be controlled by the different emitted light of DC‐CNDs depending on the excitation wavelength. ChR2 is one of the light‐switched cation channels conducting H^+^, Na^+^, K^+^, and Ca^2+^ ions.^36^ When ChR2 absorbs blue light, it opens rapidly to generate a large permeability for cations with the conformational change.^37^ We transfected the DNA plasmid enclosing ChR2 with a tag protein of yellow fluorescent protein (YFP) into HEK293T cells with lipofectamine. **Figure** **4**a shows the bright fluorescence of YFP on the confocal microscopic images to reflect the successful transfection of the plasmid with ChR2 into HEK293T cells. Both the control cells without ChR2 and ChR2‐introduced cells were incubated with 0.1 mg mL^−1^ of DC‐CNDs@PSMA solution for 1 h and then with the calcium indicator of Rhod‐2 acetoxymethyl ester (AM). Figure 4b shows the confocal microscopic images of DC‐CNDs and calcium indicators with or without laser illumination at 405 nm to compare the red fluorescence intensity in the seven spots of region of interest. The control cells treated with DC‐CNDs@PSMA showed the blue fluorescence of DC‐CNDs under 405 nm laser illumination. The ChR2‐introduced cells treated with DC‐CNDs@PSMA showed the slight blue fluorescence of DC‐CNDs and the significantly increased red fluorescence (Figures 4b,c), reflecting the elevated calcium level by the opening of ChR2 under the blue light emitted from DC‐CNDs. Because blue light cannot be generated even under laser irradiation without the treatment of DC‐CNDs, the ChR2‐introduced cells showed only slight increase in the intensity of calcium indicators as a background signal. From the results, we could confirm the feasibility of DC‐CNDs as a fluorophore to mediate the optogenetic control of neurons for the treatment of neuronal diseases. Using DC‐CNDs, we can noninvasively deliver light of two different wavelengths to the neuron by injecting the single fluorescent agent and activating it with different wavelength lasers. Thus, further optogenetic applications are expected using DC‐CNDs as an alternative for invasive LED implantation to achieve the light‐guided therapy.

**Figure 4:**
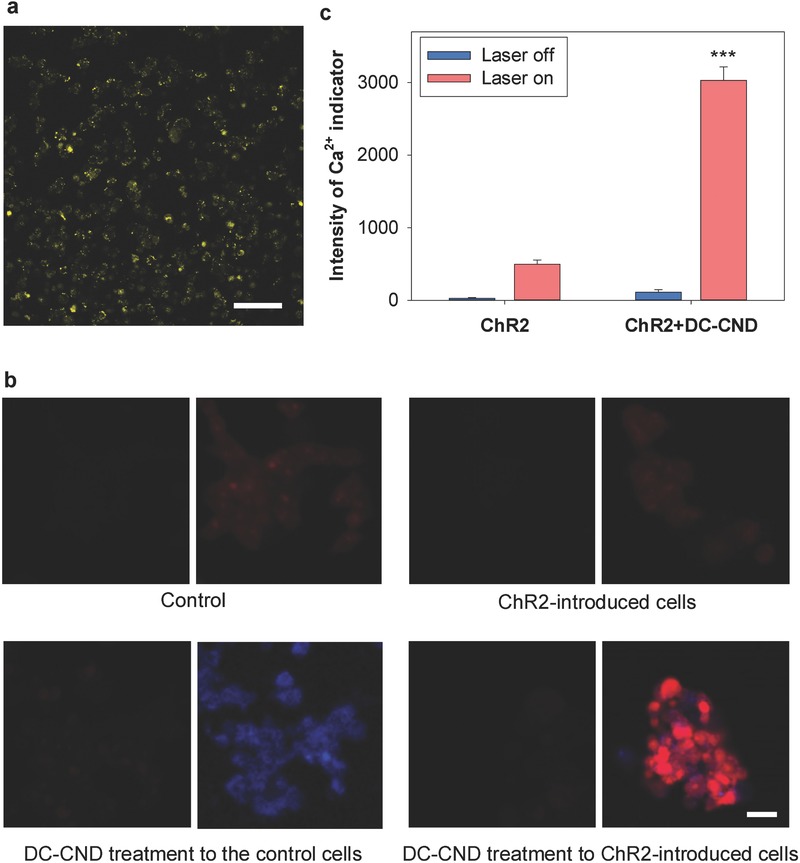
a) Confocal microscopic image to show the bright fluorescence of tag yellow fluorescent protein (YFP), reflecting the successful transfection of the plasmid with channel rhodopsin‐2 (ChR2) into HEK293T cells (scale bar = 100 µm). b) Confocal microscopic images of ChR2‐introduced HEK293T cells before (left) and after (right) illumination of 405 nm laser to show the dual‐color‐emitting carbon nanodots (DC‐CNDs) and the changes in the calcium indicator intensity after optogenetic activation of ChR2 (blue: DC‐CND, red: calcium indicator, scale bar = 25 µm). c) The quantification of calcium indicator intensity in (b) (***P < 0.001 vs ChR2‐introduced cells without DC‐CND treatment).

Finally, we assessed in vivo acute toxicity of DC‐CNDs using the collected blood samples and the dissected major organs 48 h after intravenous injection of DC‐CNDs@PSMA. According to the blood biochemistry study, the important hepatic indicators of total protein, ALB, total bilirubin, alkaline phosphate did not show the sign of liver injury as shown in **Figure** **5**a. In addition, the normal level of blood urea nitrogen and creatinine indicated the normal renal function of kidney and liver. Histological analysis of the dissected organs revealed no pathological changes or lesions in the lung, liver, kidney, and spleen (Figure 5b). These results confirmed the biocompatibility of DC‐CNDs without causing the acute toxicity at the tested dose for further in vivo biomedical applications.

**Figure 5:**
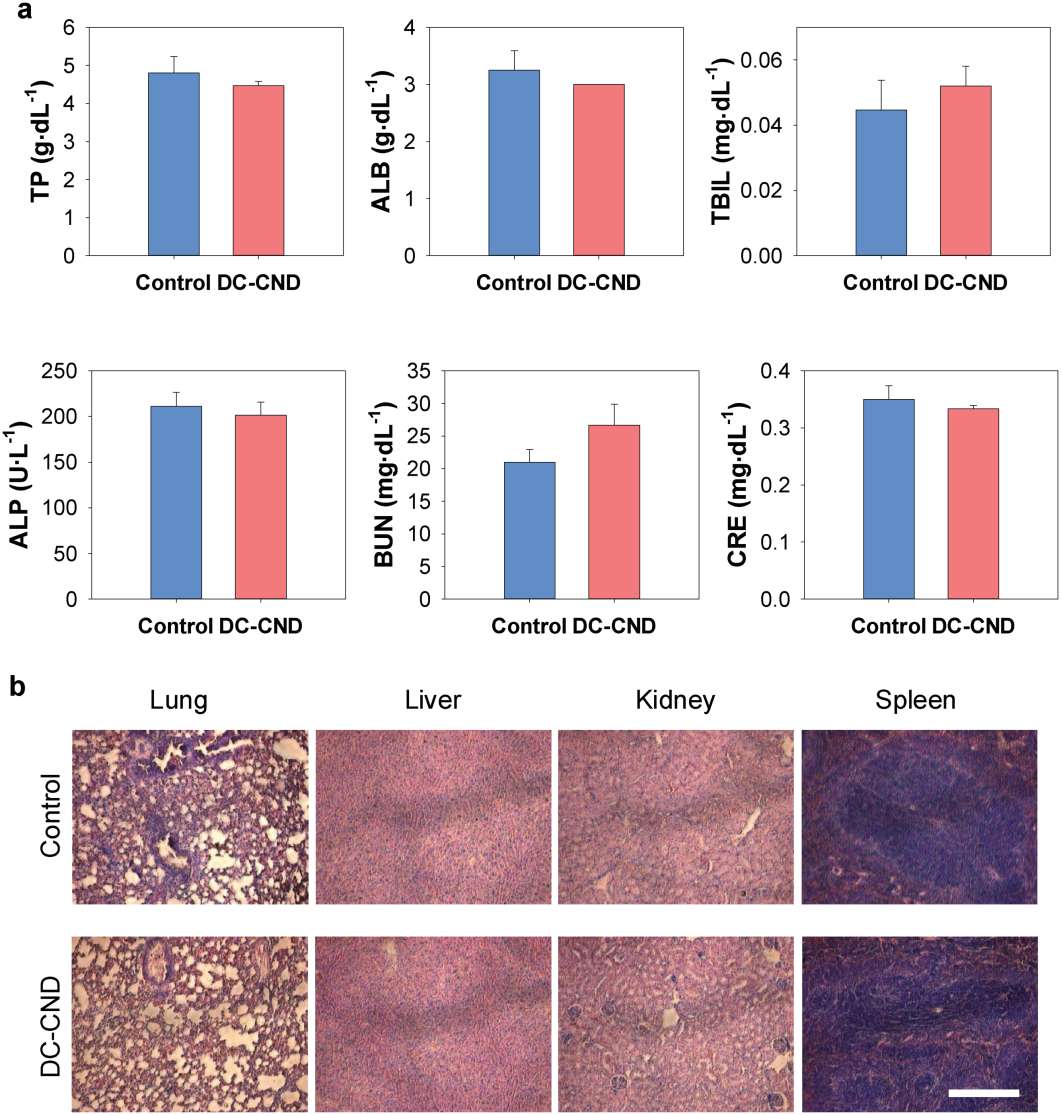
In vivo safety of dual‐color‐emitting carbon nanodots encapsulated in poly(styrene‐*co*‐maleic anhydride) (DC‐CNDs@PSMA) assessed by a) the blood biochemistry and b) the histological analysis (scale bar = 100 µm).

In conclusion, water‐soluble DC‐CNDs with two distinct excitation and emission centers have been successfully developed via the facile surface modification with 4‐octyloxyaniline. The two emission centers showed no spatial overlap and the quantum yield of the red emission center showed the highest value among red‐emissive CNDs. The DC‐CNDs showed the minimal cytotoxicity and demonstrated the great potential as a bioimaging agent for multicolor bioimaging, two‐photon microscopy, and in vivo optical imaging. Moreover, DC‐CNDs could be successfully exploited for optogenetic applications via the wavelength‐dependent activation of ion channels. Taken together, we could confirm the feasibility of DC‐CNDs with the unique characteristics of multicolor‐emitting, facile and robust preparation, and biocompatibility for various biomedical applications.

## Conflict of Interest

The authors declare no conflict of interest.

## Supporting information

Supplementary
