# Supplementary material for: Dual‐Color‐Emitting Carbon Nanodots for Multicolor Bioimaging and Optogenetic Control of Ion Channels
Source: Adv Sci (Weinh). 2017 Oct 3;4(11):1700325. doi: 10.1002/advs.201700325 (PMC5700631; doi:10.1002/advs.201700325)
Supplement: Supplementary file 1 — Supplementary [file ADVS-4-na-s001.pdf]

Copyright WILEY-VCH Verlag GmbH & Co. KGaA, 69469 Weinheim, Germany, 2017.

ADVANCED SCIENCE
Open Access

Supporting Information

for *Adv. Sci.,* DOI: 10.1002/advs.201700325

Dual-Color-Emitting Carbon Nanodots for Multicolor Bioimaging and Optogenetic Control of Ion Channels

Hyemin Kim, Yoonsang Park, Songeun Beack, Seulgi Han, Dooyup Jung, Hyung Joon Cha, Woosung Kwon, and Sei Kwang Hahn**

Copyright WILEY-VCH Verlag GmbH & Co. KGaA, 69469 Weinheim, Germany, 2017.

Supporting Information

Dual-Color-Emitting Carbon Nanodots for Multicolor Bioimaging and Optogenetic Control of Ion Channels

Hyemin Kim, Yoonsang Park, Songeun Beack, Seulgi Han, Dooyup Jung, Hyung Joon Cha, Woosung Kwon, and Sei Kwang Hahn**

Experimental Section

Materials. All chemicals were purchased from Sigma-Aldrich (St. Louis, MO) and Tokyo Chemical Industry (Tokyo, Japan). Dulbecco's modified Eagle medium (DMEM), fetal bovine serum (FBS), antibiotic-antimycotic solution, Opti-MEM I reduced serum medium, Rhod-2 acetoxymethyl ester (Rhod-2 AM), carboxyl quantum dots and Lipofectamine 2000 transfection reagent were obtained from Thermo Fisher Scientific (Waltham, MA). Antifade mounting medium was purchased from Vector Laboratories (Burlingame, CA) and a plasmid of pAAV-EF1a-double floxed-hChR2(H134R)-EYFP-WPRE-HGHpA was kindly provided by Karl Deisseroth (Addgene plasmid # 20298). SKH-1 hairless mice were purchased from Orient Bio (Seoul, Korea). All chemicals were used without further purification.

Synthesis of dual-color-emitting carbon nanodots (DC-CNDs). First, citric acid (1 g) and 0.5 M nitric acid (1 mL) were added to water (1 mL). Once citric acid was dissolved completely, the solution was added to the mixture of oleylamine (1 mL) and 1-octadecene (9 mL) in three-neck round-bottom flask. The mixture was allowed to stir for 30 min and heated for carbonization at 250°C for 3 h. After cooling to room temperature, the dark solution was

purified by centrifugation with methanol three times and dried in a vacuum oven at 80°C overnight to yield original CNDs. For surface modification, 4-octyloxyaniline (1.1 g, 5 mmol) was added to the original CNDs (15 mg) dissolved in toluene (3 mL). The solution was heated at 100°C for 12 h with vigorous stirring. After cooling to room temperature, the remaining solid was dissolved in toluene (3 mL) and then dialyzed against toluene for at least 3 days by using Spectra/Por Biotech Cellulose Ester dialysis tubes (100-500 Da). The solution was evaporated in a rotary evaporator to yield organic-soluble DC-CNDs. To control the surface modification degree, the amount of 4-octyloxyaniline was varied from 0.1 to 5 mmol.

Preparation of PSMA-encapsulated DC-CNDs (DC-CNDs@PSMA). For the polymer encapsulation, DC-CNDs (2 mg) and poly(styrene-*co*-maleic anhydride) (PSMA, 10 mg) were added to tetrahydrofuran (THF, 5 mL). After sonication for 1 h, 1 mL of aliquot was added to a mixture of ethylenediamine (10 μL) and water (2 mL). After 1 min, the mixture was mildly heated (60°C) to evaporate THF and dialyzed against water for at least 24 h to prepare DC-CNDs@PSMA. The polymer encapsulation process could be conducted with the same volume of ethanolamine instead of ethylenediamine.

Characterization of DC-CNDs. Transmission electron microscopy (TEM) was performed by using Jeol JEM-2200FS equipped with a Cs corrector. X-ray photoelectron spectroscopy (XPS) was performed by using an Escalab 250 spectrometer with an Al X-ray source (1486.6 eV). ^{13}C nuclear magnetic resonance (NMR) spectra of CND and DC-CND dissolved in $CDCl_3$ and ^{1}H NMR spectrum of DC-CNDs@PSMA dissolved in D_2O were recorded on a Bruker DRX600 spectrometer (600 MHz). Fourier-transform infrared (FT-IR) spectroscopy were recorded on a Nicolet iS50 FT-IR spectrometer. UV-vis absorption and photoluminescence (PL) spectroscopy were performed with 10 mm × 10 mm QS-grade quartz cuvettes (Hellma Analytics 111-QS). The light absorption spectra were recorded on a Scinco S-3100 spectrophotometer. The PL spectra were recorded on a Jasco FP-8500 fluorometer. The quantum yield was measured with 2 mm × 10 mm QS-grade quartz cuvettes (Jasco Parts

Center 6808-H250A). The absolute quantum yields were recorded on a Jasco FP-8500 fluorometer equipped with a 100 mm integrating sphere setup (ILF-835) and calculated by using Jasco Spectra Manager II Software.[1] The transient absorption spectroscopy was performed by using a visible Helios system (Ultrafast systems) having a 1 kHz femtosecond Ti:sapphire laser system (Libra) with 780 nm center wavelength and 80 fs pulse duration. The laser beam was divided by a beam splitter. About 95% of the laser beam drove an optical parametric amplifier (TOPAS Prime) as a tunable pump beam (250–560 nm) with the pulse duration of 200 fs. The other 5% was focused on a 5-mm sapphire crystal to generate a white-light continuum as a probe beam (400–750 nm). The time-resolved photoluminescence (TRPL) spectroscopy was performed by using the same laser setup. The data were recorded on a Princeton Instrument SP2300 spectrometer combined with a Hamamatsu C5680 streak camera. In the spectroscopy of UV-vis, PL, TA and TRPL, the solvents were toluene for DC-CNDs and water for DC-CNDs@PSMA, respectively.

Cytotoxicity assessment of DC-CNDs@PSMA. Mouse hepatocytes (FL83B), human hepatocellular carcinoma cells (HepG2), mouse skin melanoma cells (B16F10) and human embryonic kidney cells (HEK293) cells were cultured in high glucose DMEM supplemented with 10 vol% FBS and 10 $IU·mL^{-1}$ of antibiotic-antimycotic solution. The cytotoxicity of DC-CNDs@PSMA in various kinds of cells was assessed by MTT assay. The cells were suspended at a concentration of 1×10^5 $cells·mL^{-1}$ in the medium and 100 μL of the cell suspension containing 1×10^4 cells was seeded on the each well of 96-well flat bottom cell culture plate. On the next day, the medium was replaced with 100 μL of fresh medium containing DC-CNDs@PSMA at a concentration range from 0.125 to 2 $mg·mL^{-1}$. Carboxyl quantum dots dissolved in the medium at the same concentrations were used to compare the cytotoxicity. The cells were incubated at 37°C in a humidified 5% CO_2 cell culture incubator for 24 h. Then, MTT reagent was added to each well and incubated at 37°C in the cell culture incubator for 1 h. DMSO (50 μL) was added to dissolve the formazan in each well and the

optical density was measured at 540 nm using a microplate reader (Molecular Devices, Sunnyvale, CA). Four replicates were performed for the experiment.

In vitro bioimaging of DC-CNDs@PSMA. B16F10, HEK293, FL83B and HepG2 cells were suspended at a concentration of 1×10^5 cells·mL^{-1} in the medium, and 200 μL of the cell suspension containing 2×10^4 cells was seeded on the 8-chamber confocal slide. On the next day, the medium was replaced with 200 μL of fresh medium containing 0.5 mg·mL^{-1} of DC-CNDs@PSMA and incubated for 1 h. Then, the cells were washed with PBS thrice, fixed with 4 wt% paraformaldehyde in PBS, washed again with PBS thrice, and observed with Leica TCS SP5II MP SMD FLIM (Leica, Deerfield, IL) at a magnification of ×250. The DC-CNDs@PSMA were excited with a diode laser at 405 nm, a diode-pumped solid-state (DPSS) laser at 561 nm, and a titanium-sapphire laser at 950 nm. The emission light of DC-CNDs@PSMA was spectrally resolved into different channels.

In vivo bioimaging of DC-CNDs@PSMA. The DC-CNDs@PSMA solution (100 μL, 1 mg·mL^{-1}) in PBS was administered to 6-week old SKH-1 hairless mice via tail-vein injection. At the predetermined time intervals, the fluorescence signal from the mice treated with DC-CNDs was observed with an optical imaging system (IVIS; Caliper Life Sciences, Hopkington, MA). The photonic emission was assessed with Living Image 4.1 Software (PerkinElmer, Waltham, MA). The data were shown by pseudocolor representation for light intensity. All animal experiments were performed following the laboratory animal protocol approved by the institutional animal care and use committee of the Pohang University of Science and Technology in accordance with the National Institutes of Health Guide for the Care and Use of Experimental Animals.

In vivo acute toxicity test of DC-CNDs@PSMA. After optical imaging for 48 h, the animals were sacrificed to collect the major organs and blood to carry out the preliminary acute toxicity test. The major organs of lung, liver, kidney and spleen were fixed in 4% paraformaldehyde, embedded in paraffin, sectioned, and stained with hematoxylin and eosin

(H&E). The blood chemistry was analyzed using a chemistry analyzer (BS-380, Mindray Medical International Limited, Shenzhen, China).

Preparation of optogenetic cell line. HEK293T cells were transfected using a DNA plasmid of pAAV-EF1a-double floxed-hChR2(H134R)-EYFP-WPRE-HGHpA to express channelrhodopsin-2 (ChR2) as described elsewhere.[2] HEK293T cells were cultured in high glucose DMEM supplemented with 10 vol% FBS without antibiotic-antimycotic solution. The cells were suspended at a concentration of 1×10^5 cells·mL^{-1} in the medium and 300 μL of the cell suspension containing 3×10^4 cells was seeded on the poly(L-lysine)-coated coverglass-bottom confocal dish. On the next day, the medium was replaced with Opti-MEM I reduced serum medium. The DNA-Lipofectamine complex was prepared by incubating the mixture of 1 μg DNA plasmid, 4 μL Lipofectamine 2000, and 200 μL Opti-MEM I medium at room temperature for 20 min. The DNA-Lipofectamine complex solution (50 μL) was added to the cells and incubated at 37°C in a humidified 5% CO_2 cell incubator for a day. The transfection was assessed by confocal imaging of yellow fluorescent protein (YFP) which is a fluorescence tag for visualization.

Calcium imaging to visualize light-gated ChR2 activation. The light-gated ChR2 activation in the DC-CNDs@PSMA-treated cells was examined by calcium imaging before and after excitation of DC-CNDs@PSMA. The ChR2-introduced cells were incubated with 0.1 mg·mL^{-1} of DC-CNDs@PSMA solution for the intracellular uptake in antibiotics-free DMEM for 1 h. Then, the cells were washed with PBS thrice and the medium was replaced with Tyrode's solution for the treatment with the calcium indicator of Rhod-2 AM. Rhod-2 AM (50 μg) was dissolved in 44.5 μL of DMSO to prepare the stock solution, and 5 μL of the stock solution was mixed with 5 μL of 40 mg·mL^{-1} Pluronic solution and 1 mL of Tyrode's solution. Then, the Rhod-2 mixture was treated to the cells at room temperature for 20 min followed by the aspiration of the remaining mixture. The cells were washed with PBS thrice and incubated with Tyrode's solution for calcium imaging. Calcium imaging was carried out

by using Leica TCS SP5II MP SMD FLIM with a DPSS laser at 561 nm. The DC-CNDs@PSMA-treated cells were excited with a diode laser at 405 nm, and the intensity of the calcium indicator before and after the excitation of DC-CNDs@PSMA was compared to investigate the light-gated activation of ChR2. The detection range of channel was set narrow and the power of the laser was set low to minimize the interference from the fluorescence signal of DC-CNDs@PSMA on the intensity of calcium indicator. The calcium level was determined in average by quantifying the intensities of calcium indicator of seven selected spots of region of interest (ROI).

References

[1] C. Würth, M. Grabolle, J. Pauli, M. Spieles, U. Resch-Genger. *Nat. Protoc.* **2013**, *8*, 1535

[2] V. Chan, D. M. Neal, S. G. M. Uzel, H. Kim, R. Bashir, H. H. Asada, *Lab Chip.* **2015**, *15*, 2258.

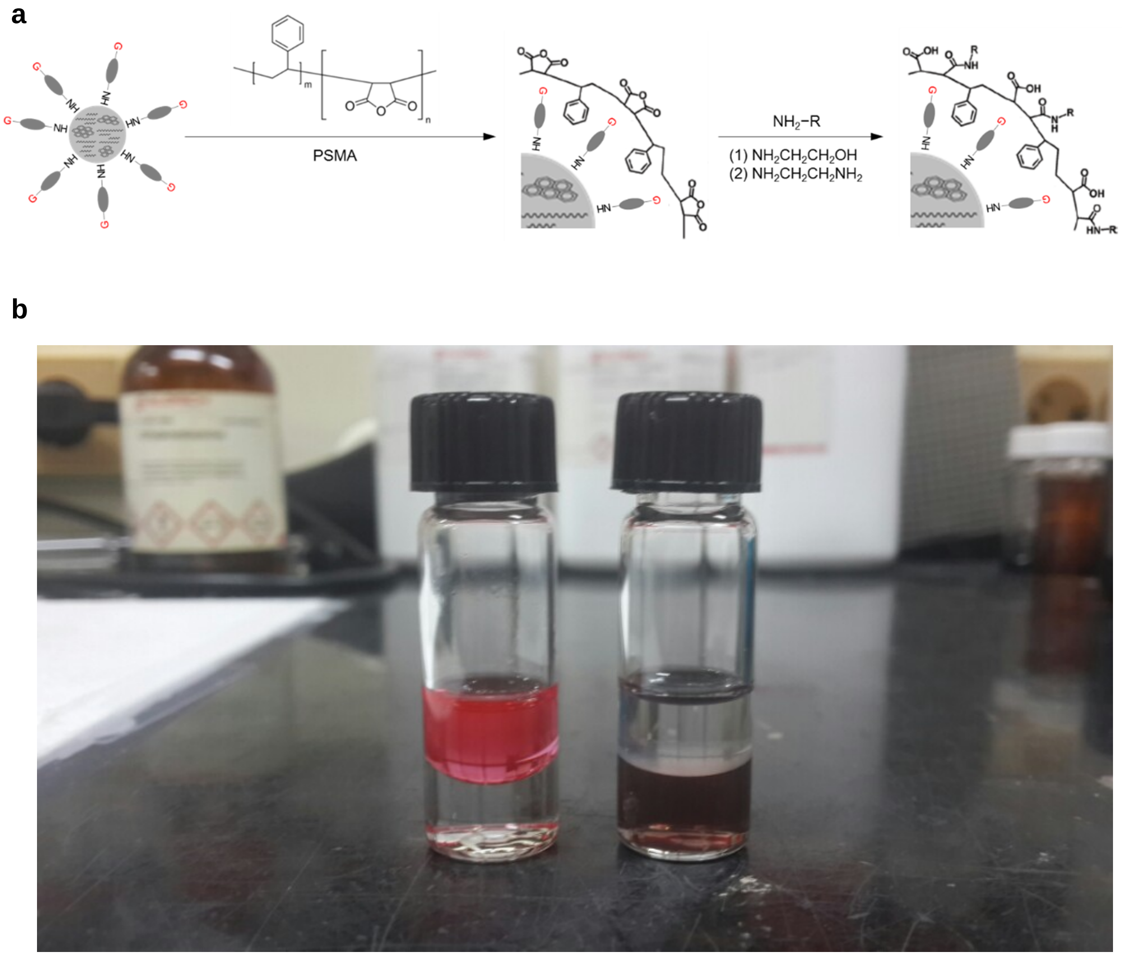

Figure S1. (a) Schematic representation for the preparation of DC-CNDs@PSMA. (b) The photoimage of DC-CNDs@PSMA before (left) and after (right) the polymer encapsulation. The upper and lower phases are toluene and water phases, respectively.

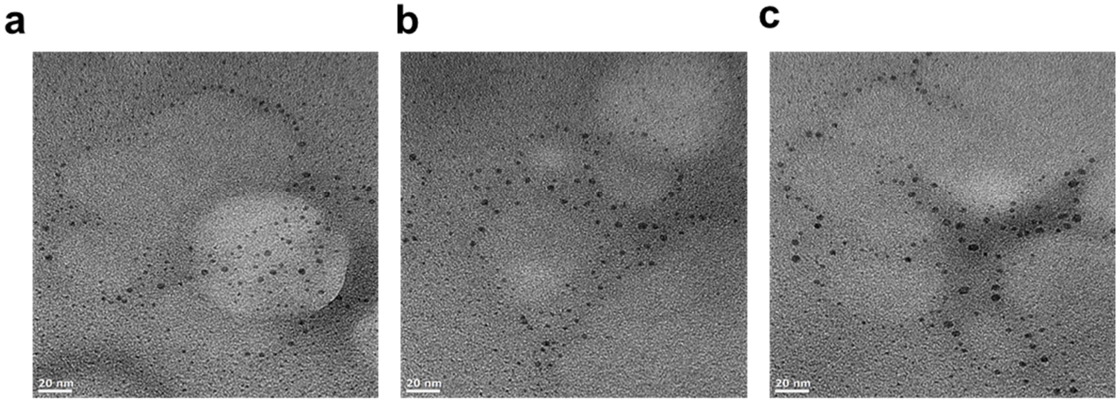

Figure S2. (a-c) Low magnification TEM images of DC-CNDs@PSMA at different regions (scale bar = 20 nm).

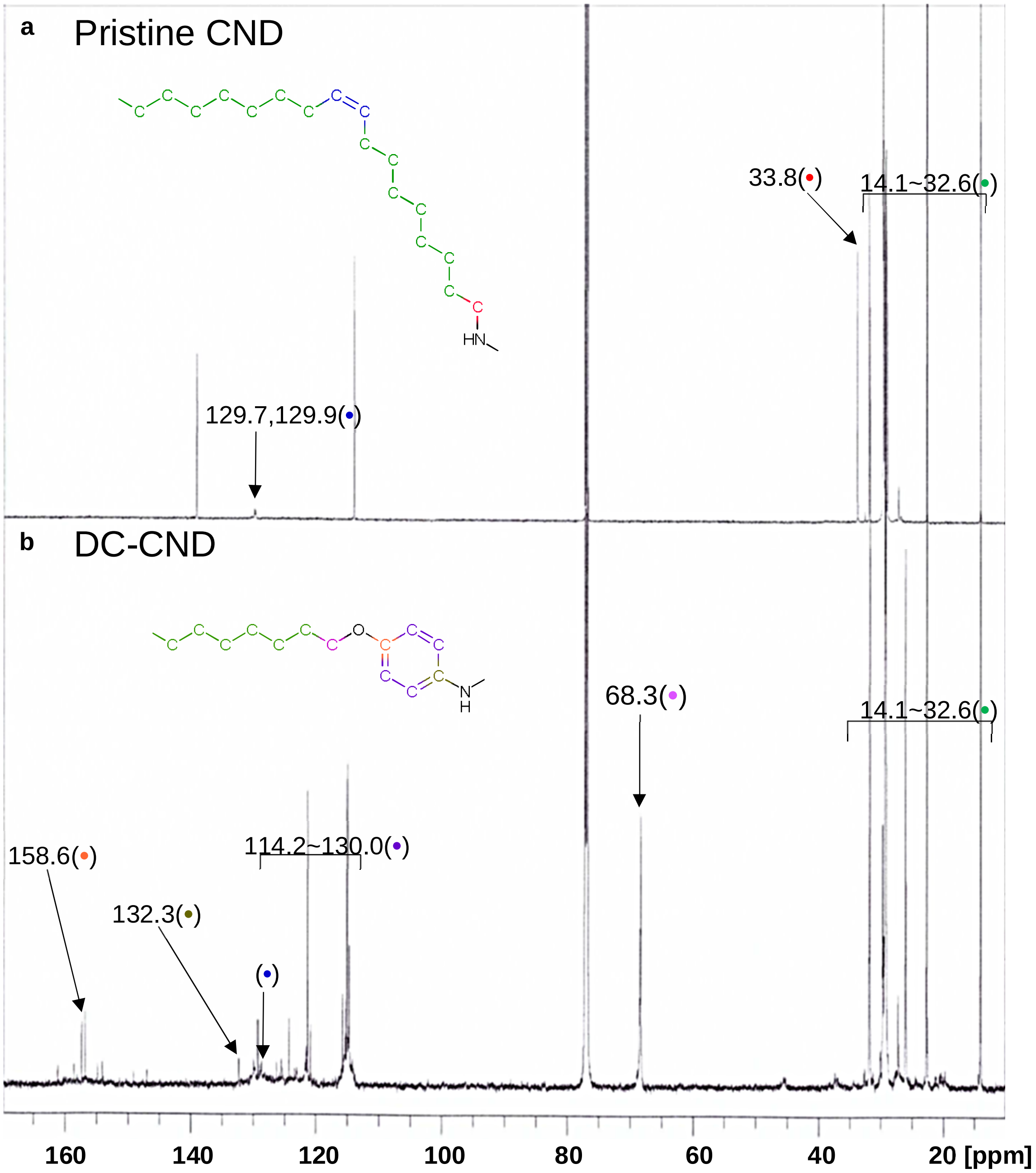
a
Pristine CND
HN
129.7,129.9(•)
33.8(•)
14.1~32.6(•)
b
DC-CND
O
N
H
68.3(•)
14.1~32.6(•)
158.6(•)
114.2~130.0(•)
132.3(•)
(•)
160
140
120
100
80
60
40
20 [ppm]

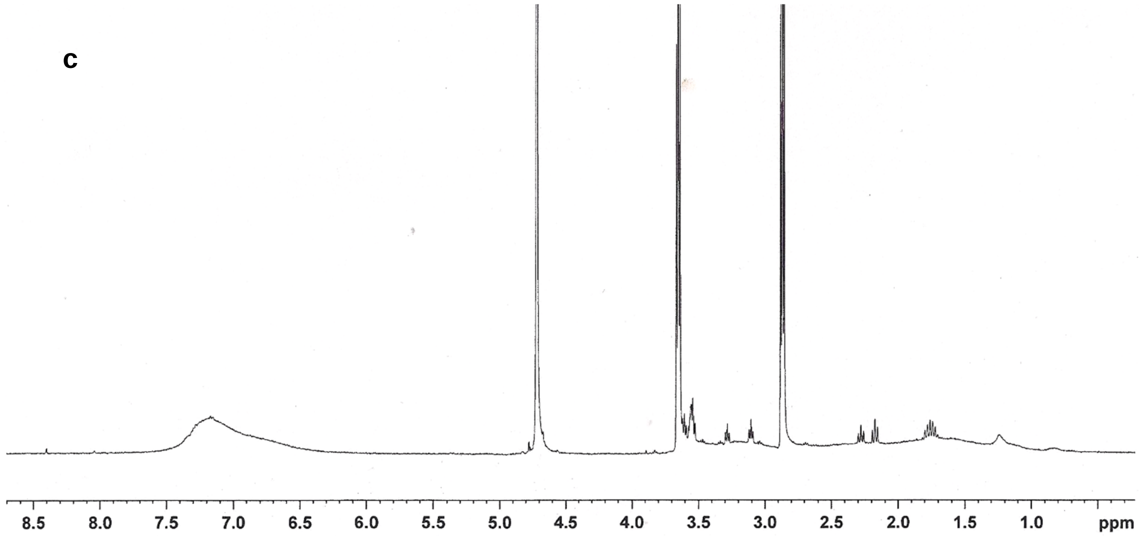

Figure S3. ^{13}C nuclear magnetic resonance (NMR) data of (a) pristine CND and (b) DC-CND. The insets show the chemical structure of surface-modified molecules. Color coding represents chemical bondings. (c) ^{1}H NMR data of DC-CNDs@PSMA.

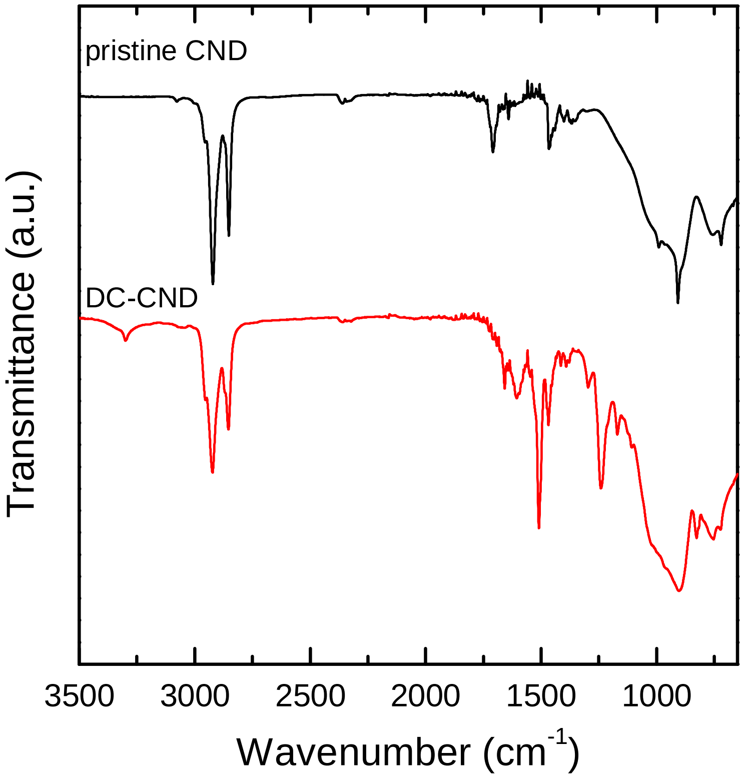

Figure S4. FT-IR spectra of pristine CND and DC-CND.

NOTE : The C–H stretching (≈2950-2800 cm^{-1}), C=O stretching (≈1690 cm^{-1}), N–H bending (≈1500 cm^{-1}), C=C stretching (≈1600-1450 cm^{-1}) and =C–H bending (990 cm^{-1} for trans and 760 cm^{-1} for cis) bands commonly found in pristine CNDs and DC-CNDs showed that the surface carbonyl groups and passivated oleylamine were presented in our CNDs. After surface modification, the N–H stretching (3500-3300 cm^{-1}) and C–O stretching (1260 cm^{-1}) peaks were developed and the N–H bending and C=C stretching (1600-1450 cm^{-1}) bands were intensified due to the chemical bonding of 4-octyloxyaniline to the surface of CNDs.

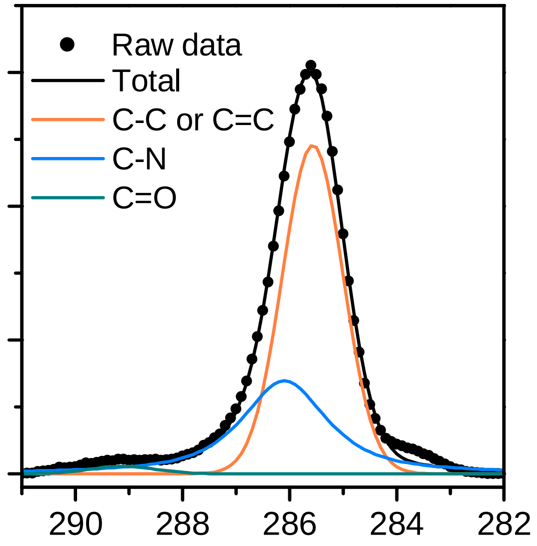

Figure S5. Deconvoluted carbon (1s) XPS spectra of pristine CNDs.

Table S1. The elemental analysis data of pristine CNDs and DC-CNDs.

Sample	Atomic ratio (%)		
	C1s	N1s	O1s
Prinstine CND	89.38	2.25	8.37
DC-CND	79.28	7.01	13.69

NOTE : The deconvoluted C1s XPS spectra indicated the presence of C-C/C=C (285.4 eV), C-N (286.1 eV), and C=O (289.1 eV) bondings on the surface of CNDs, likely due to oxidative carbonization with nitric acid, and passivation with oleylamine.

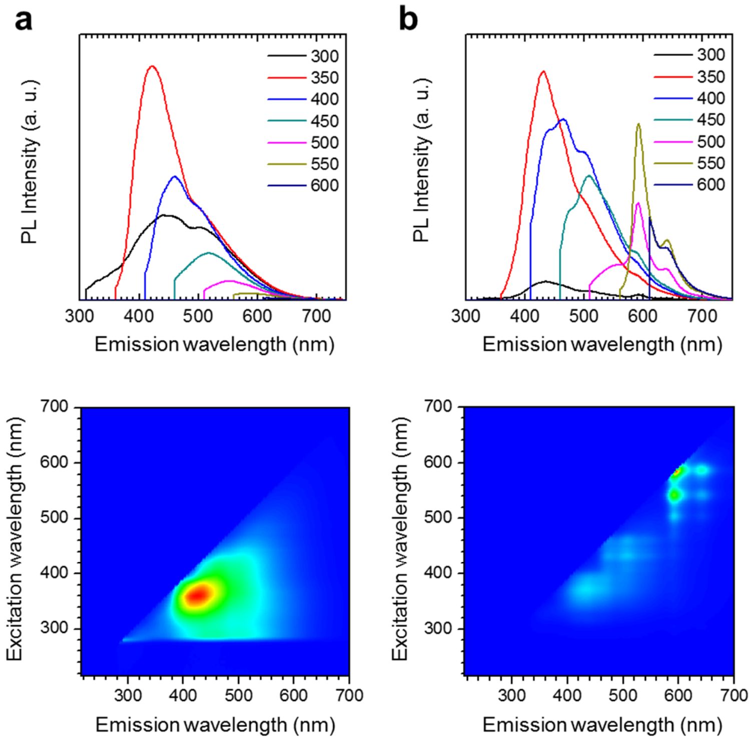

Figure S6. Excitation dependent emission spectra (up) and PL contour maps (down) of (a) pristine CNDs and (b) DC-CNDs.

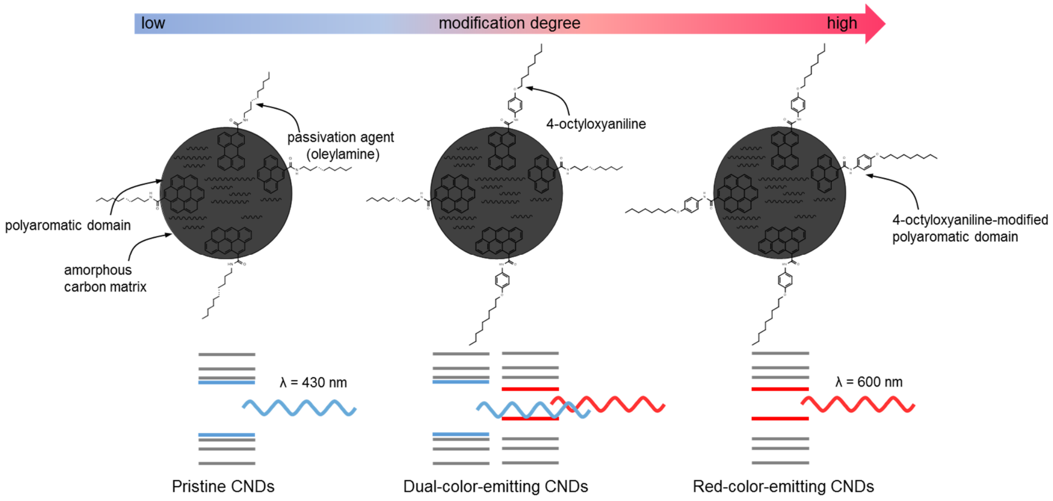

Figure S7. Chemical and electronical structure changes of CNDs according to degree of surface modification.

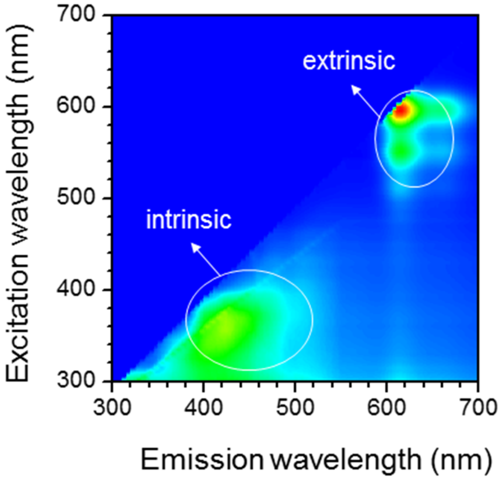

Figure S8. PL map of DC-CNDs@PSMA. The used solvent was water.

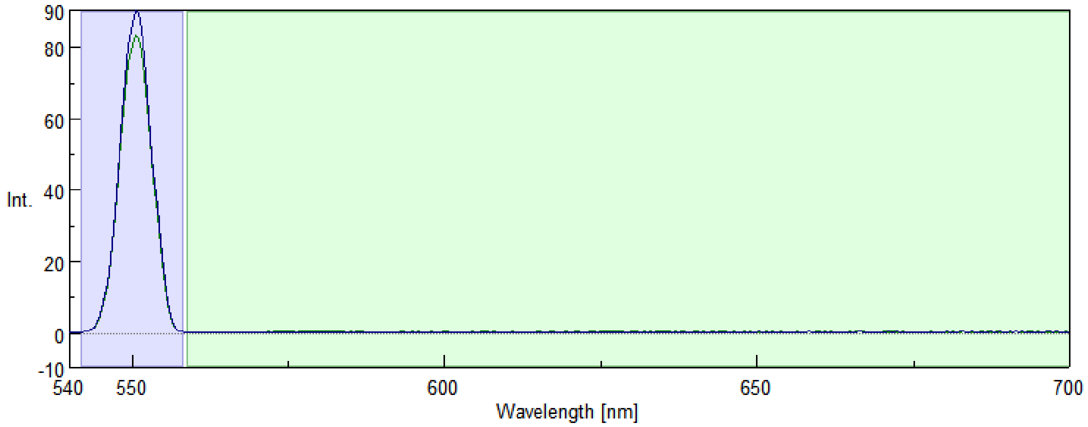

	File Name	Absorptance (%)	Quantum Efficienc	Quantum Efficiency	Incident Light	Fluorescence	Scattering	Inc
1	O.qydt	5.836	1.784	30.560	537.75	9.59	506.37	

Figure S9. The quantum yield calculation of DC-CNDs by using the Jasco Spectra Manager II Software.

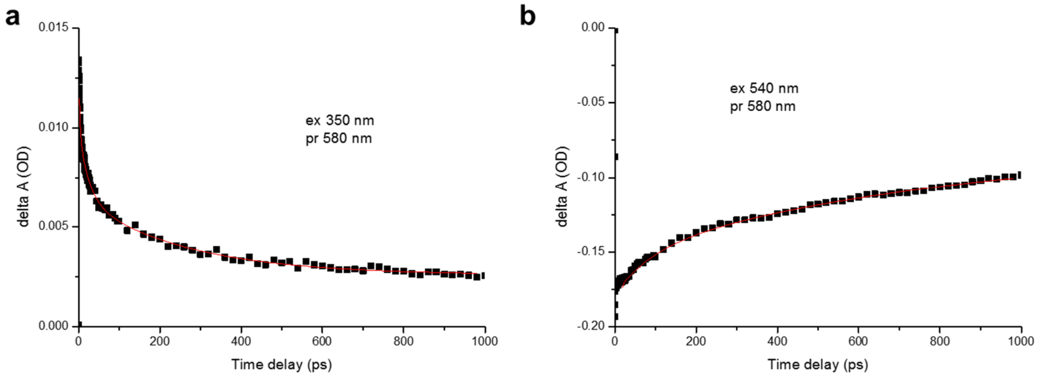

Figure S10. The time-resolved absorption (TA) spectra of DC-CNDs at the excitation wavelengths of (a) 350 nm and (b) 540 nm. The wavelength of probe light was 580 nm. The data were fitted to bi-exponential functions to calculate the decay times (red lines).

Table S2. The decay times of photoexcited electrons in DC-CNDs.

Sample	Origin	Ex wavelength (nm)	Probe wavelength (nm)	τ_1 (ps)	τ_2 (ps)
DC-CND	Intrinsic	350	580	33.12	367.59
	Extrinsic	540	580	96.04	1276.17

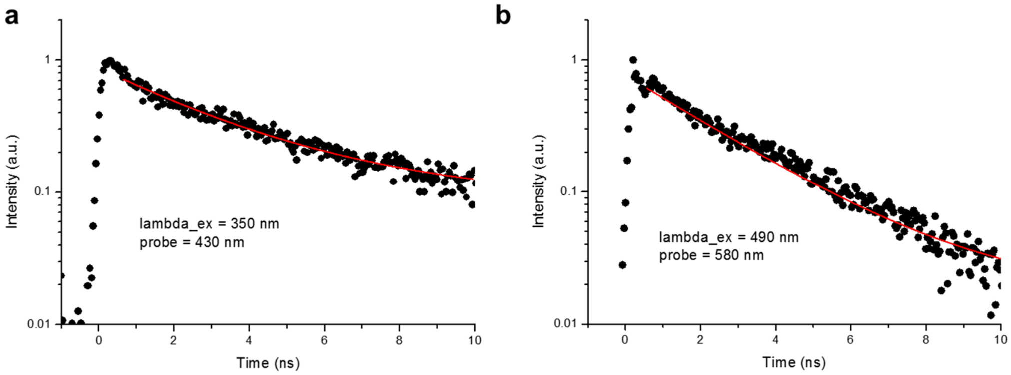

Figure S11. The time-resolved photoluminescence (TRPL) spectra of DC-CNDs at the excitation wavelengths of (a) 350 nm and (b) 490 nm, respectively. The data were fitted to the single exponential functions to calculate the photoluminescence lifetimes (red lines). The legends show the wavelengths of probe light.

Table S3. The photoluminescence lifetimes of DC-CNDs.

Sample	Origin	Ex wavelength (nm)	Probe wavelength (nm)	Lifetime (ns)
DC-CND	Intrinsic	350	430	1.9
	Extrinsic	490	580	3.1

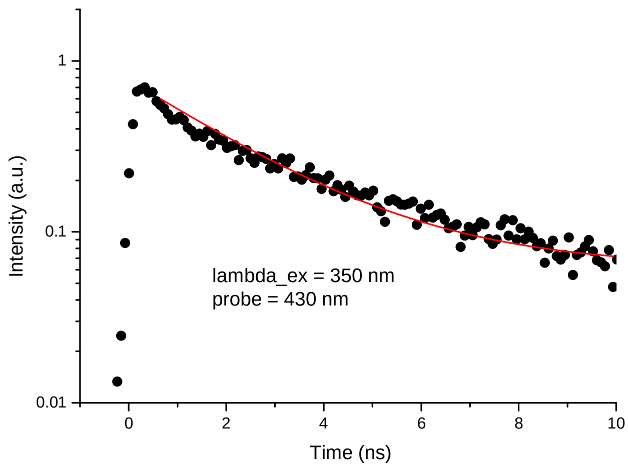

Figure S12. The time-resolved photoluminescence (TRPL) spectra of pristine CNDs at the excitation wavelengths of 350 nm. The data were fitted to the single exponential functions to calculate the photoluminescence lifetimes (red lines). The legend shows the wavelength of probe light.

Table S4. The photoluminescence lifetime of pristine CNDs.

Sample	Origin	Ex wavelength (nm)	Probe wavelength (nm)	Lifetime (ns)
Pristine CND	Intrinsic	350	430	2.4

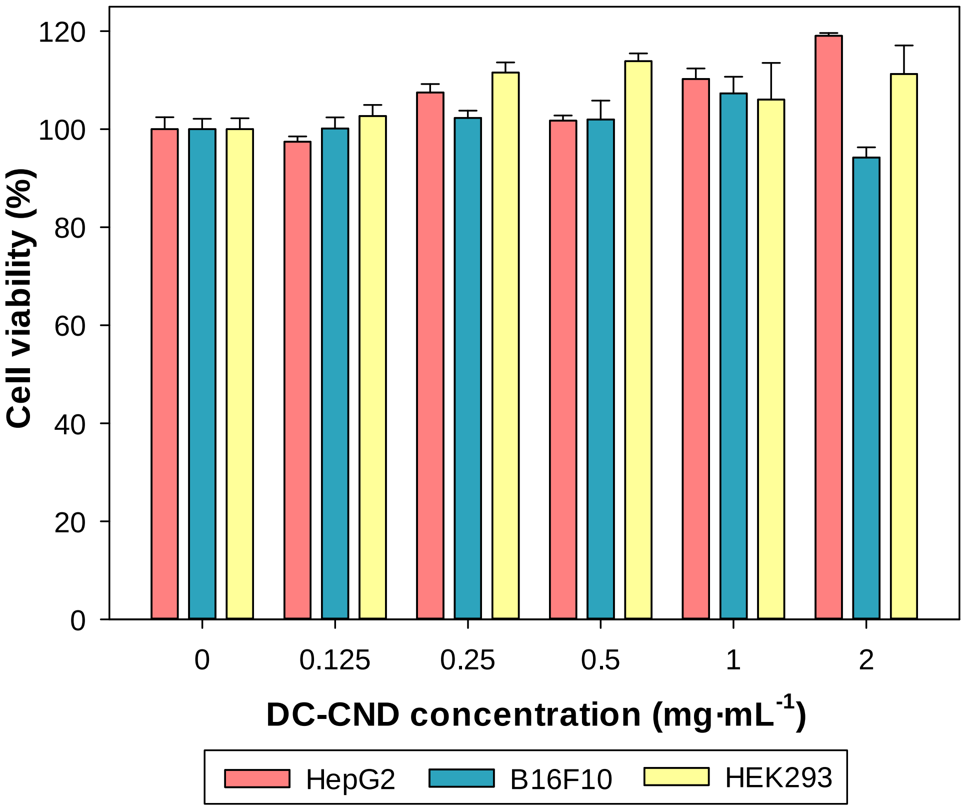

Figure S13. The cytotoxicity of DC-CNDs in HepG2, B16F10, and HEK293 cells.

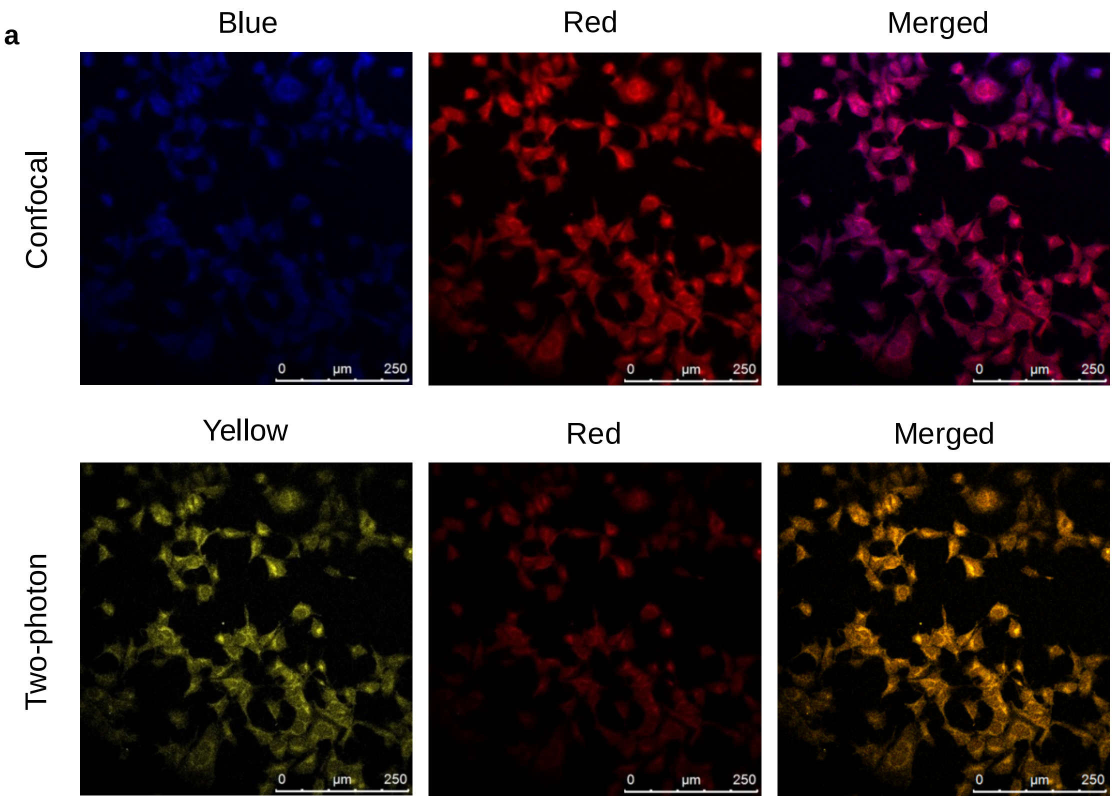
a
Blue
Red
Merged
Confocal
Yellow
Red
Merged
Two-photon
0 μm 250

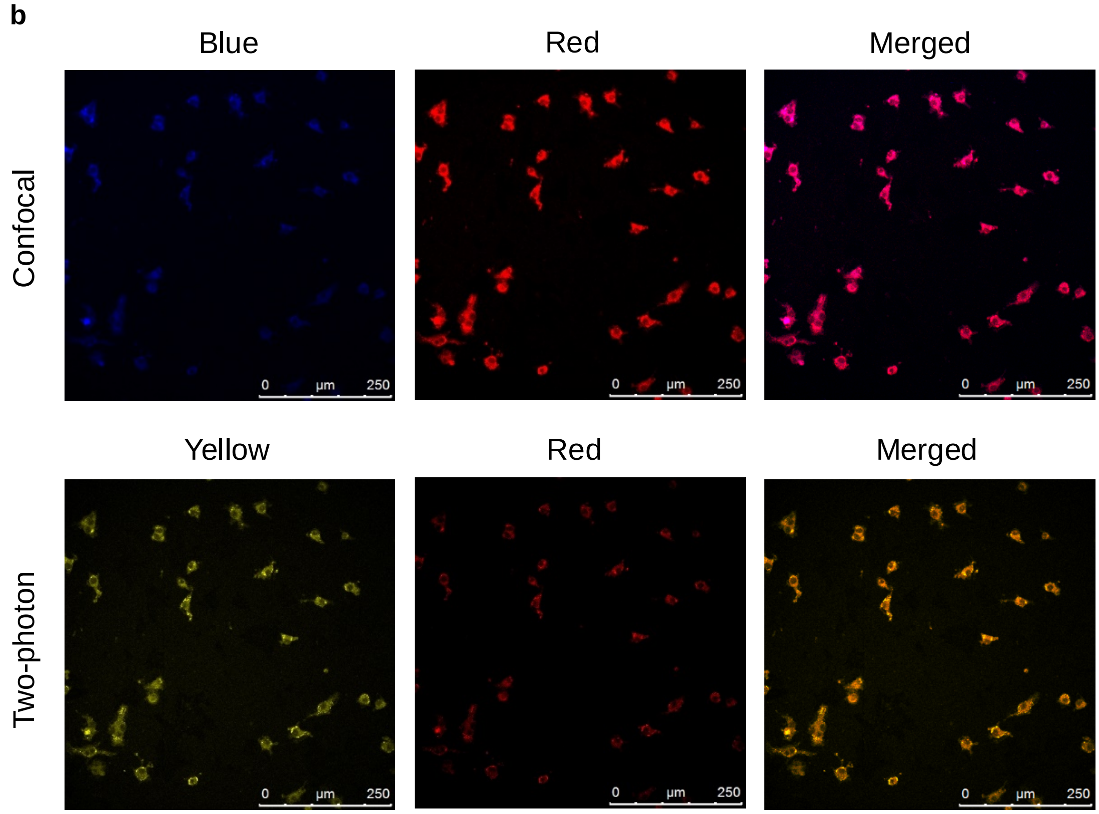
b
Blue
Red
Merged
Confocal
Yellow
Red
Merged
Two-photon
0 μm 250

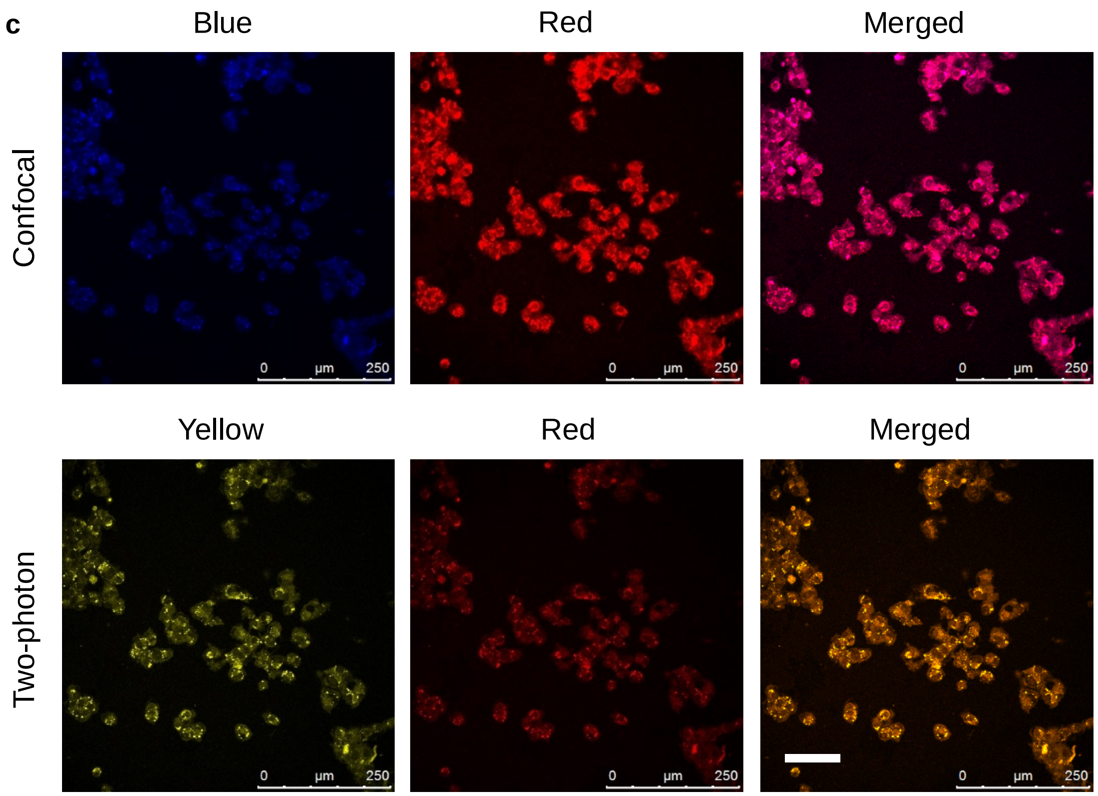

Figure S14. Multicolor confocal (top) and two-photon (bottom) images of (a) HEK293, (b) FL83B and (c) HepG2 cells treated with DC-CNDs (scale bar = 100 μm).
